# Association between chronic health problems and quality of life in medical students. Results of the POLLEK cohort study

**DOI:** 10.3389/fpubh.2026.1824586

**Published:** 2026-05-29

**Authors:** Szymon Szemik, Angelina Kaleta-Pilarska, Joanna Kowalska, Małgorzata Kowalska

**Affiliations:** Department of Epidemiology, School of Medicine in Katowice, Medical University of Silesia in Katowice, Katowice, Poland

**Keywords:** chronic disease, follow-up study, health status, medical students, quality of life

## Abstract

**Introduction:**

Medical students with chronic diseases constitute a particurarly vulnerable subgroup of young adults, exposed to various health risks, related both to the academic environment and their underlying health condition. Previous findings suggest that medical students with chronic illnesses experience greater deterioration in quality of life (QoL) compared with their healthy peers. The aim of this study was to validate earlier observations from the POLLEK study regarding the relationship between quality of life, health status, and the prevalence of chronic diseases among medical students during a two-year follow-up.

**Material and methods:**

This study presents results from the final cohort of Polish medical students' longitudinal study (POLLEK), recruited during the academic years 2020/2021-2022/2023 at the Medical University of Silesia in Katowice. A total of 887 first-year students (T1) participated in the baseline assignment, and 705 were followed up in the second year of studies (T2). The questionnaire included measures of quality of life (WHOQOL-BREF), hazardous alcohol use (AUDIT), general health status (GHQ-28), lifestyle indicators, and sociodemographic characteristics. Students were divided into two groups: those with previously diagnosed chronic diseases (DCD) and those without declared chronic diseases (NDCD).

**Results:**

Among first-year students (T1), 219 (24.7%) reported ever having a diagnosed chronic disease. In the second academic year (T2), this proportion increased to 28.9% (*N* = 204). Higher scores for overall QoL (*p* < 0.001), and in the somatic (*p* = 0.009), psychological (0.006), and environmental (*p* = 0.028) domains were observed among first-year students without chronic diseases. Similar patterns were observed during the second academic year, except for the psychological domain (*p* = 0.782).

**Conclusions:**

Chronic diseases are relatively common among medical students and are associated with poorer quality of life, worse self-rated health, and lower psychological wellbeing in the early years of medical education. Students with chronic diseases report consistently lower quality of life, more somatic complaints, and higher levels of anxiety and depressive symptoms, with these differences persisting over time. These findings highlight the need for systematic monitoring of students' wellbeing and early, tailored support, including health-focused interventions, coping support, and appropriate academic accommodations.

## Introduction

Chronic diseases represent a major global health challenge, especially in countries with older populations. The World Health Organization (WHO) reported that it accounts for the majority of deaths worldwide, with tens of millions of fatalities each year (1). Approximately 20% of school-age youth in the United States live with a chronic medical condition, and about one-third of these students experience sequelae severe enough to interfere with school participation regularly (2). Cited studies indicate that students with chronic medical conditions are less likely to graduate from high school on time compared to their healthy peers. Students with chronic conditions often face feelings of frustration, isolation, sadness, and anger. Additionally, they frequently struggle with fatigue and concentration, further complicating their ability to attend classes (3). Medical students represent a unique subgroup of young adults who face multiple challenges. They are exposed to various health risk factors, including those related to unhealthy lifestyles (4, 5), psychological aspects (6, 7), and the academic environment (8, 9). These factors are well-known determinants of chronic disease development and can lead to deterioration in quality of life in later life. While most students maintain good health during their studies, some evidence suggests that chronic diseases occur within this population (8), including mental health issues such as depression and burnout, which can manifest as early as the first year of study (9).

The concept of quality of life (QoL) revolves around the balance between individuals' goals, expectations, standards, concerns, and their perception of their life situation within the framework of their culture and value system (10). This multidimensional idea encompasses both positive elements, such as role functioning, contentment, and mobility, and negative aspects, including negative emotions, reliance on medication, fatigue, and pain. Notably, the presence of chronic diseases, especially comorbidity, is often associated with a lower quality of life (11).

Current published data indicate that chronic diseases can have a long-term impact on many dimensions of quality of life (QoL), including pain, anxiety, depression, sleep quality, happiness, stress levels, and occupational functioning (10). In particular, the psychological aspect appears to be a significant factor in assessing QoL among youth. Additionally, adolescents with chronic diseases often experience an identity crisis, which further deteriorates their quality of life (13, 14). A large population-based study conducted by Wang et al. found that approximately half of adolescents and young adults with chronic health problems reported experiencing moderate to severe impacts on their QoL (11).

The arguments presented above, along with the limited evidence in the bibliography regarding the relationship between chronic diseases and quality of life (QoL) in medical students, make it difficult to draw clear conclusions. Our previous publication suggests that medical students with diagnosed chronic health problems tend to report lower QoL compared with their healthy peers, and these disparities may persist or even worsen over time (12). These findings underscore the need to continue longitudinal studies aimed at examining both the prevalence of chronic diseases in this population and their long-term effects on QoL and overall health status, as explored in the presented study. Expanding the sample size and enabling tracking of observations of the same students over time would significantly enhance the credibility of the evidence crucial for effective prevention. By addressing these concerns, we can foster a healthier and more resilient generation of medical professionals.

## Material and methods

### Participants and design

The paper presents results from the final cohort of Polish medical students (POLLEK) recruited during the academic years 2020/2021 and 2022/2023 at the Medical University of Silesia in Katowice. A total of 887 medical students were involved in the first year of studies (T1) and, subsequently, 705 in the second year of studies (T2). All students declared written consent to participate in the study. The project was granted by the Medical University of Silesia (No. BNW-1-021/K/5/I).

### The questionnaire

The POLLEK questionnaire included standardized questions regarding the assessment of quality of life (WHOQOL-BREF), the prevalence of hazardous alcohol use (AUDIT), as well as questions about health status (self-declared health, occurrence of chronic diseases including type of health conditions), lifestyle indicators (i.e., tobacco smoking, selected eating behaviors, and physical activity), and socio-demographics (age, sex, marital status, current financial situation, and place of residence during study). The paper version of the questionnaire was administered by lecturers and completed by students during scheduled classes. A detailed description of each used questionnaire is available in our previous publications (13, 14). The current paper presents findings from an additional questionnaire used in the study, the General Health Questionnaire-28 (GHQ-28). This questionnaire consists of 28 questions designed to evaluate overall psychological wellbeing. It is organized into four subscales, with each subscale containing seven items: somatic symptoms, anxiety/insomnia, social dysfunction, and severe depression. Participants responded to the questions using a Likert scale that ranged from 0 to 3 points. Overall scores and particular scores for each subscale were calculated. Finally, the disease names reported by the participants were assigned to groups defined according to the ICD-11 classification.

### Statistical analysis

Categorical variables were presented as a number and percentage of observations (*N*; %). Next, the differences of categorical variables for selected subgroups were tested by the chi-squared test. Quantitative variables are presented as mean values with standard deviations. Differences between specific subgroups were assessed using the Mann-Whitney U test due to the non-normal distribution of the variables. Multiple regression models were employed to assess the factors influencing the Quality of Life (QoL) and General Health Questionnaire (GHQ) domains separately during the T1 and T2 periods. This analysis was conducted for two groups: those with previously diagnosed chronic diseases (DCD) and those without declared chronic diseases (NDCD). A *p*-value of less than 0.05 was considered statistically significant for all analyses. Any missing values that hindered the calculation of the regression models were automatically excluded from the analysis.

## Results

The study group in the first year of observation (T1) included 887 medical students, and in the second year, 705. It was noted that 183 people dropped out of the study, mainly due to a lack of progress in their studies, transfer to another academic center, or a lack of consent to continue the study. Among first-year students, 219 (24.7%) declared the occurrence of ever-recognised chronic disease. In the second academic year, the percentage of students with chronic diseases increased to the value of 28.9% (*N* = 204). Detailed data are presented in Table 1.

**Table 1:** Number and percentage of students with declared and no declared chronic disease (DCD and NCDC, respectively) in both years of observation, according to selected explanatory variables.

Variable		Study period			
		T1 (1st year; 2021/2022) *N* = 887		T2 (2nd year; 2022/2023) *N* = 705	
		DCD	NDCD	DCD	NDCD
		*N* (%)	*N* (%)	*N* (%)	*N* (%)
Sex	Women	154 (26.1)	435 (73.9)	153 (31.9)	327 (68.1)
	Men	65 (21.8)	233 (78.2)	51 (22.7)	174 (77.3)
*p-value*		0.157		**0.011**	
Marital status	In relationship	67 (28.6)	167 (71.4)	75 (34.7)	141 (65.3)
	Single	147 (23.0)	492 (77.0)	122 (25.7)	353 (74.3)
*p-value*		0.086		**0.014**	
Current financial situation	Poor	68 (26.4)	178 (73.6)	62 (36.5)	108 (63.5)
	Good	154 (23.9)	490 (76.1)	140 (26.3)	393 (73.7)
*p-value*		0.435		**0.010**	
Current place of residence during studies at university	Family home	46 (20.4)	179 (79.6)	35 (22.0)	124 (78.0)
	Dormitory/rented flat or room	173 (26.1)	490 (73.9)	168 (30.9)	375 (69.1)
*p-value*		0.089		**0.029**	
Current cigarette smoking (traditional or electronic)	Yes	36 (31.0)	80 (69.0)	20 (31.2)	44 (68.8)
	No	183 (23.7)	588 (76.3)	183 (28.7)	455 (72.3)
*p-value*		0.089		0.665	
Hazardous alcohol use	Hazard use	68 (24.8)	206 (75.2)	65 (31.9)	139 (68.1)
	Low risk	136 (24.3)	424 (75.7)	120 (26.5)	332 (73.5)
*p-value*		0.866		0.161	
Number of meals containing animal protein	More than 75% of daily meals	197 (25.3)	580 (74.7)	115 (25.9)	329 (74.1)
	Less often	21 (19.3)	88 (80.7)	88 (33.8)	172 (66.2)
*p-value*		0.166		**0.024**	
Consumption of fruit and vegetables	More than 2 meals daily	150 (25.0)	449 (75.0)	144 (29.9)	337 (70.1)
	Less often	69 (24.1)	217 (75.9)	58 (26.1)	164 (73.9)
*p-value*		0.767		0.299	
Frequency of physical activity	High	58 (23.7)	187 (76.3)	65 (27.2)	174 (72.8)
	Low	161 (25.1)	481 (74.9)	138 (29.8)	325 (70.2)
*p-value*		0.664		0.470	
Self-rated health	Worse	140 (37.0)	238 (63.0)	109 (39.5)	165 (60.5)
	Good	79 (15.5)	430 (84.5)	94 (22.0)	334 (78.0)
*p-value*		**< 0.0001**		**< 0.0001**	

N, number; DCD, declared chronic disease; NDCD, students without chronic diseases; T1, the first year of study; T2, the second year of study; p, statistical significance in chi-square test.

The percentage of first-year medical students with previously recognized chronic diseases was significantly higher in those with weaker self-declared health status, 37.0% vs. 15.5%, respectively (*p* < 0.0001). However, the results of re-evaluation in the second year of studies revealed that the frequency of declared chronic diseases is significantly higher in students with a poorer financial situation (*p* = 0.010), in relationship (*p* = 0.014), not living in a family home (*p* = 0.029), not preferring animal protein in each meals (0.024), and also with the worst self-declared health status (*p* < 0.0001).

What is important, the list of chronic diseases is long, from the most frequent declared asthma, allergies, including atopic dermatitis (*n* = 78; 8.7%), thyroid disease (*n* = 28; 3.1%), metabolic diseases, including diabetes (*n* = 24; 2.7%), depression, neurosis, and anxiety states (*n* = 18; 2.0%), diseases of the musculoskeletal system (*n* = 14; 1.6%), polycystic ovary syndrome or other hormonal disorders (*n* = 10; 1.2%), cardiovascular diseases (*n* = 6; 0.7%), migraines (*n* = 6; 0.7%), visual defects (*n* = 5; 0.6%), albinism (*n* = 3; 0.3%), epilepsy (*n* = 2; 0.2%), autoimmune disease (*n* = 2; 0.2%), acne (*n* = 2; 0.2%), to single cases of leukemia, Lyme disease, kidney stones, Asperger's syndrome, endometriosis, psiorasis, Reyno's syndrome, phenyloketonuria, anemia, polyneuropathy, systemic inflamation. Moreover, it was noted that the number of first-year medical students with one chronic disease was 157 (17.6% of all subjects), but 52 (5.8%) had at least two diseases. Ten students did not list the name of their declared chronic disease.

The obtained results (Table 2) confirmed that higher scores in total QoL (*p* < 0.001), somatic (*p* = 0.009), psychological (0.006), or environmental domains (*p* = 0.028) were associated with first-year medical students without recognized chronic diseases. During the second academic year, the observations were similar, except in the psychological domain (*p* = 0.782). Additionally, significant differences were not found between the DCD and NDCD groups in terms of social relationships in both academic years.

**Table 2:** WHOQOL-BREF standardized scores (mean value) in medical students classified by the occurrence of declared chronic diseases (DCD) and non-declared chronic diseases (NDCD), separately at the T1 and T2 periods.

QoL domain	The first year of observation T1(2021/2022)		The second year of observation T2(2022/2023)	
	DCD (*N* = 217)	NDCD (*N* = 666)	DCD (*N* = 204)	NDCD (*N* = 496)
	M (SD)	M (SD)	M (SD)	M (SD)
Overall Qol	61.1 (19.3)	69.7 (17.3)	62.9 (19.0)	70.8 (16.8)
	*p* < 0.001		*p* < 0.001	
Somatic	49.4 (15.8)	53.0 (17.8)	46.4 (13.4)	44.5 (12.1)
	*p* = 0.009		*p* = 0.040	
Psychological	57.9 (16.8)	61.9 (14.7)	55.7 (13.9)	55.6 (13.3)
	*p* = 0.006		*p* = 0.782	
Social relationships	69.5 (20.6)	68.9 (18.5)	67.2 (22.4)	70.7 (40.9)
	*p* = 0.365		*p* = 0.406	
Environmental	62.7 (13.2)	65.2 (12.4)	64.4 (13.6)	67.7 (12.9)
	*p* = 0.028		*p* = 0.001	

M, mean; SD, standard deviation; *N*, number; *p*, statistical significance in the *U* Mann-Whitney test; DCD, Declared Chronic Disease; NCDC, No Declared Chronic Disease.

**Table 3:** GHQ scores in groups of medical students classified by the occurrence of declared chronic disease (DCD) or non-declared chronic disease (NDCD), separately at the T1 and T2 periods.

GHQ domain	The first year of observation T1(2021/2022)		The second year of observation T2(2022/2023)	
	DCD (*N* = 217)	NDCD (*N* = 666)	DCD (*N* = 204)	NDCD (*N* = 496)
	M (SD)	M (SD)	M (SD)	M (SD)
Overall GHQ	35.7 (13.9)	32.1 (12.8)	32.4(15.7)	28.9(12.0)
	*p* = 0.002		*p* = 0.002	
Somatic	9.3 (4.1)	8.1 (3.8)	9.1(4.3)	8.1(3.7)
	*p* = 0.0003		*p* = 0.0002	
Anxiety	11.1 (4.1)	10.3 (4.5)	10.0(5.3)	9.0(4.5)
	*p* = 0.028		*p* = 0.001	
Social	9.5 (3.8)	9.1 (3.5)	8.6(4.0)	8.3(3.2)
	*p* = 0.282		*p* = 0.755	
Depression	5.6 (5.3)	4.4 (4.5)	4.8(5.2)	3.4(4.0)
	*p* = 0.004		*p* = 0.005	

M, mean; SD, standard deviation; N, number; p, statistical significance in the *U* Mann-Whitney test; DCD, Declared Chronic Disease; NCDC, No Declared Chronic Disease.

Additionally, the findings from the multiple regression model indicated that self-rated health status, current financial situation, and self-reported chronic disease significantly affected overall quality of life in both years of observation (Supplementary material 1).

Results of the GHQ questionnaire suggest that statistically lower scores in overall, somatic, anxiety, and depression domains revealed medical students without recognized chronic diseases. This observation was similar for both academic years, T1(2021/2022) and T2 (2022/2023).

Moreover, the findings from the multiple regression model indicated that poorer self-rated health status and female sex were significantly related to higher GHQ scores in both years of observation (Supplementary material 2).

It was noted that the relationship between overall QoL and GHQ scores was rather moderate and statistically significant, regardless of whether the analysis was conducted for the entire group or separately for students with or without a diagnosed chronic disease, in both years of observation. Values of Spearman correlation coefficient were R = −0.49; *p* < 0.001 (total subjects), R = −0.53; *p* < 0.001 (students with chronic diseases), R = −0.47; *p* < 0.001 (students without chronic disease) in the first year of observation. The results for the second year of study were as follows: R = −0.54; *p* < 0.001, R = −0.61; *p* < 0.001, and R = −0.52; *p* < 0.001, respectively, in total, DCD and NDCD students.

## Discussion

The main objective of this study was to validate the previously published observation regarding the relationship between quality of life and health status and the occurrence of chronic diseases among medical students in a two-year follow-up study. Research for the POLLEK project is ongoing, with complete results now available for the full cohort. Recruitment began in the 2021/2022 academic year and was concluded in 2022/2023. The final group of subjects consisted of 887 first-year medical students, while in the second year, there were only 705 students. Our findings showed that the prevalence of diagnosed chronic diseases (DCD) was consistent with previously published data for the incomplete cohort. The percentage of DCD was 23.7% (*N* = 101) (12) and now (in the completed cohort) is 24.7% (*N* = 219) among first-year medical students. Similarly, among second-year students, the frequency of DCD was 28.3% for the incomplete cohort and 28.9% of students (*N* =204) for the completed cohort. Finally, only 5.8% of students (*n* = 52) in the first year reported at least two diseases. These findings align with an analysis of Portuguese medical students, which found that 23% reported having a chronic disease (15). The authors of the cited work noted that their results closely align with the 25.7% prevalence found in the national population aged 25 to 34. In contrast, research conducted among medical students in Morocco showed a substantially higher prevalence of chronic disease, reaching 41.5% of the study population (16). Moreover, evidence from a systematic review and meta analysis including 183 studies conducted across 43 countries worldwide demonstrated that the global prevalence of depression or depressive symptoms among medical students was 27.2%, while the prevalence of suicidal ideation reached 11.1% (17). Then, the prevalence of burnout syndrome among medical students worldwide has been shown to be highly heterogeneous, ranging from 5.6 and 88% (18). Additionally, national data from Poland further indicate that one-quarter of adults report having a chronic condition, with prevalence steadily increasing with age. Among individuals up to 29 years old, the most commonly reported chronic condition is allergies, which affect 9% of this age group (19).

The study also aimed to verify key factors affecting the quality of life and health status in medical students. In our completed cohort, students with diagnosed chronic diseases reported significantly lower overall quality of life and worse functioning in all specific domains, such as physical health, social functioning, and daily activities, compared to their healthy peers. This pattern aligns with findings from another study of university students, which identified chronic pain and mental disorders as independent factors that predict a decline in health-related quality of life. The authors emphasize the importance of early identification of poor quality of life, mental health issues, and chronic conditions to facilitate effective treatment (20).

Additionally, the negative effects of chronic conditions went beyond just physical or functional limitations. Our study confirmed a decline in psychological wellbeing, which was reflected in increased somatic symptoms, elevated anxiety levels, and more frequent depressive symptoms. This finding aligns with broader evidence from student populations. For example, a recent meta-analysis revealed that the prevalence of depression among university students is 27%, which is strongly linked to a lower quality of life (QoL) (21) Furthermore, the prevalence of depression among medical students is approximately 28% globally, with higher rates observed among female students and those in their first year of medical school (22).

The study we conducted involved the same individuals at two time points: T1 (2021/2022) and T2 (2022/2023). It found that students who reported having a chronic illness consistently rated their overall quality of life, health status, and psychological wellbeing as worse than their peers at both measurement periods. This persistent effect over time suggests that chronic illness may impose a stable and possibly increasing burden on quality of life and perceived general health. These longitudinal findings align with other research highlighting the importance of providing ongoing support to medically ill students throughout their education (23, 24).

Several mechanisms may explain the observed associations, particularly regarding the decline in quality of life and its persistence over time. Firstly, chronic conditions often lead to symptoms such as ongoing pain, fatigue, physical limitations, or a weakened immune system. Given the demands of medical studies, including long hours of classes, clinical duties, and academic stress, these symptoms can significantly impact daily functioning, treatment, and participation in demanding activities. Secondly, medical students may differ in their coping strategies and ability to manage. Individual resources and health factors play a crucial role in how students respond to stress. These differences can also stem from various life experiences, family backgrounds, the availability of social support, and individual cognitive and motivational abilities (25). What is more, personality characteristics may play an important role in shaping coping styles in response to chronic disease (26). Previous studies have demonstrated associations between personality among medical students and their choice of medical specialty (27). Research has explored the relationship between personality traits and medical students' vulnerability and anxiety-related disorders and hypochondriacal tendencies. Especially, health anxiety scores have been shown to correlate positively with anxious and emotionally labile personality traits (28).

Students with chronic diseases often experience higher levels of stress, poorer sleep quality, and reduced physical activity, which limits their ability to cope effectively (29). For instance, healthy students tend to have more structured daily rhythms: regular meals, regular sleep hours, and study time, which promotes better cognitive and emotional functioning. This regularity is one element of a healthy lifestyle that makes it easier to cope with academic and life demands. Students with chronic illnesses may experience more frequent interruptions in their circadian rhythm due to doctor appointments, hospitalizations, or worsening symptoms. This affects the sense of stability and causes further disruptions to their daily schedule (30, 31). As a result, they tend to have less effective coping strategies, are more likely to feel tense or exhausted, and struggle more with balancing study and relaxation compared to their healthy peers. In contrast, healthy students are more likely to adopt flexible, health-promoting strategies. They find it easier to engage in physical activity, maintain a regular sleep schedule, and organize their study time effectively. Young adults without health issues generally exhibit higher self-esteem and better social functioning, which helps protect them against stress. The differences in coping strategies between students with and without chronic diseases may exacerbate existing disparities in mental health and daily functioning (32).

Our study has significant implications for medical education and student support programs. Universities should implement systematic screening for chronic conditions, mental health symptoms, and quality of life indicators at various points throughout medical training. Early identification could lead to timely interventions, such as tailored psychological support, peer mentoring, and adjustments to academic workloads.

Additionally, it is crucial to integrate resilience-building and adaptive coping strategies into the curriculum, especially for students with chronic illnesses, to help them navigate both health challenges and academic responsibilities. For example, mindfulness-based interventions, including yoga sessions and meditation, have been shown to reduce perceived stress and enhance self-regulation, self-compassion, and empathy. Students participating in such programs also report improved stress management skills. Other effective initiatives may include psychological therapy, nutritional counseling, structured physical activity, and facilitated reflection groups, which provide opportunities for students to share experiences and foster peer support (33, 34). The development and implementation of comprehensive wellbeing programs are especially important in the Polish context, where central regulations regarding psychological support for medical students are lacking. As a result, mental support is typically organized individually by medical schools and is often limited to short-term or temporary therapeutic interventions rather than long-term, systemic solutions (35).

Finally, our findings emphasize the importance of adopting a holistic approach to student wellbeing that addresses both physical and mental health.

## Strengths and limitations

The primary strength of this study lies in its relatively high response rate, 90.5% and 88.2%, respectively, at the first (T1) and second (T2) measurement points. This strong response rate enhances the generalizability of the findings to the student population at the Medical University of Silesia in Katowice. However, it is worth noting that previously published results from an incomplete cohort were similar (12). A slight increase in the prevalence of chronic diseases was observed during the initial years of medical education, along with deterioration in students' quality of life. These findings suggest that the results are consistent and comparable even after expanding the size of the cohort. Furthermore, the data were collected longitudinally, with measurements taken at two points in time among the same group of medical students. It is important to highlight that research of this kind has not been conducted before.

The results of our study highlight a significant public health issue concerning the wellbeing of medical students who have chronic diseases, a topic that has not been extensively explored within this population. Most research on the mental health of medical students tends to focus on aspects such as stress, burnout, or general psychiatric symptoms, while the relationships between chronic conditions and student performance are often overlooked. By incorporating information about chronic disease diagnoses, quality of life, psychological symptoms, and health behaviors, this study emphasizes a complex issue that has direct implications for medical education, prevention strategies, and student support systems.

While the POLLEK study has several strengths, there are important limitations to consider. First, it is a single-center study, which restricts the ability to generalize the findings to all Polish medical students. Expanding the study to multiple centers would improve its external validity and allow for comparisons across different institutions. Second, the diagnoses of chronic diseases were based on self-reports and were not clinically verified. Although self-reporting is commonly used in epidemiological research and reflects the students' perceptions of their health, there is a possibility of misclassification or recall bias. Third, although the study was conducted longitudinally, it only included two time points over a relatively short period. Chronic diseases and their psychosocial effects can change over time, so additional follow-ups would provide a more comprehensive understanding of their long-term trajectories. The study is ongoing, and we are currently gathering information from students in their fourth and fifth years. Finally, while the study assessed a wide range of quality of life and psychological indicators, it did not include in-depth measures of coping strategies, social support, or personality traits. These factors may moderate the relationship between chronic illness and functioning, and would be valuable additions in future research. Overall, despite these limitations, the study provides important and novel evidence regarding the challenges faced by medical students with chronic health conditions, highlighting the need for targeted interventions and institutional policies aimed at improving students' wellbeing.

## Conclusions

The results of the study indicate that chronic diseases are relatively common among medical students and contribute to a significant health burden. The presence of these diseases is linked to a lower quality of life, poorer perceived health, and reduced psychological wellbeing in the early years of medical education. Additionally, medical students with chronic illnesses consistently report a lower overall quality of life and experience negative impacts across physical, psychological, and environmental domains. They also exhibit higher levels of somatic complaints, anxiety, and depressive symptoms. Notably, these disparities persist over time, suggesting that chronic illness has a lasting and potentially cumulative negative effect on wellbeing. These findings highlight the need for a more comprehensive and proactive approach to student wellbeing in medical education. Systematic monitoring of quality of life, mental health, and chronic health conditions, along with early tailored support, may help mitigate long-term negative consequences. Interventions should address physical health limitations, promote adaptive coping strategies, and implement structural accommodations within medical curricula. Special attention should be given to students with chronic diseases, as they may require more individualized academic flexibility and ongoing psychosocial support.

## Data Availability

The raw data supporting the conclusions of this article will be made available by the authors, without undue reservation.
