# Supplementary material for: Association between chronic health problems and quality of life in medical students. Results of the POLLEK cohort study
Source: Front Public Health. 2026 May 29;14:1824586. doi: 10.3389/fpubh.2026.1824586 (PMC13259856; doi:10.3389/fpubh.2026.1824586)
Supplement: Supplementary file 1 [file Table_1.docx]

**Supplement 1.** Statistically significant predictors of QoL in medical students, results of multiple regression (GRM).

| Independent Variable | Regression Coefficient (95% CI) |
| --- | --- |
| The first year of observation T1(2021/2022) | |
| Overall QoL (R^2^ =0.287, p<0.001) | |
| Current financial situation (1=poor, 2=good) | 0.076 (0.018, 0.134) |
| Self-rated health (1=poor, 2=good) | 0.476 (0.041, 0.536) |
| Declared chronic disease (1=yes, 2=no) | 0.079 (0.021, 0.138) |
| Number of meals with animal protein (1=more than 75% daily meals, 2=rare) | -0.071 (-0.130, -0.011 |
| Somatic (R^2^ = 0.139, p<0.001) | |
| Self-rated health (1=poor, 2=good) | 0.343 (0.276, 0.409) |
| Current place of residence (1=family house, 2=dormitory, rented flat or room | -0.065 (-0.128, -0.002) |
| Psychological (R^2^ = 0.160, p<0.001) | |
| Current financial situation (1=poor, 2=good) | 0.064 (0.001, 0.127) |
| Self-rated health (1=poor, 2=good) | 0.349 (0.283, 0.415) |
| Number of meals with animal protein (1=more than 75% daily meals, 2=rare) | -0.081 (-0.146, -0.015) |
| Social relationships (R^2^ = 0.134, p<0.001 ) | |
| Marital status (1=in relationship, 2=single) | -0.257 (-0.320, -0.195) |
| Current financial situation (1=poor, 2=good) | 0.083 (0.019, 0.146) |
| Self-rated health (1=poor, 2=good) | 0.221 (0.155, 0.287) |
| Environmental (R^2^= 0.204, p<0.001) | |
| Current financial situation (1= poor, 2=good) | 0.352 (0.251, 0.373) |
| Current cigarette smoking (1=yes, 2=no) | 0.083 (0.022, 0.144) |
| Self-rated health (1=poor, 2=good) | 0.239 (0.175, 0.304) |
| The second year of observation T2(2022/2023) | |
| Overall QoL (R^2^ =0.303, p<0.001) | |
| Current financial situation (1=poor, 2=good) | 0.105 (0.040, 0.169) |
| Self-rated health (1=poor, 2=good) | 0.481 (0.414, 0.547) |
| Declared chronic disease (1=yes, 2=no) | 0.09 (0.033, 0.163) |
| Somatic (R^2^ = 0.159, p<0.001) | |
| Current financial situation (1=poor, 2=good) | 0.207 (0.136, 0.278) |
| Frequency of physical activity (1=high, 2=low) | -0.106 (-0.177, -0.034) |
| Self-rated health (1=poor, 2=good) | 0.249 (0.175, 0.322) |
| Declared chronic disease (1=yes, 2=no) | -0.137 (-0.208, -0.065) |
| Psychological (R^2^ = 0.045, p<0.001) | |
| Sex (1=woman, 2=man) | -0.093 (-0.166, -0.020) |
| Current financial situation (1=poor, 2=good) | 0.082 (0.008, 0.155) |
| Self-rated health (1=poor, 2=good) | 0.170 (0.096, 0.245) |
| Social relationships (R^2^ = 0.039, p=0.001 ) | |
| Marital status (1=in relationship, 2=single) | -0.099 (-0.174, -0.024) |
| Current financial situation (1=poor, 2=good) | 0.097 (0.021, 0.173) |
| Environmental (R^2^= 0.235, p<0.001) | |
| Sex (1=woman, 2=man) | 0.083 (0.015, 0.151) |
| Current financial situation (1=poor, 2=good) | 0.385 (0.318, 0.451) |
| Self-rated health (1=poor, 2=good) | 0.199 (0.132, 0.266) |
| Number of meals with animal protein (1=more than 75% daily meals, 2=rare) | -0.082 (-0.150, -0.014) |

Legend: CI, Confidence Interval. R^2^, determination of the model. p, the significance of the multivariable regression model.
