# Supplementary material for: Association between chronic health problems and quality of life in medical students. Results of the POLLEK cohort study
Source: Front Public Health. 2026 May 29;14:1824586. doi: 10.3389/fpubh.2026.1824586 (PMC13259856; doi:10.3389/fpubh.2026.1824586)
Supplement: Supplementary file 2 [file Table_2.docx]

**Supplement 2.** Statistically significant predictors of GHQ in medical students, results of multiple regression (GRM).

| Independent Variable | Regression Coefficient (95% CI) |
| --- | --- |
| The first year of observation T1(2021/2022) | |
| Overall GHQ (R^2^ =0.208, p<0.001) | |
| Sex (1=woman, 2=man) | -0.194 (-0.256, -0.131) |
| Self-rated health (1=poor, 2=good) | -0.339 (-0.404, -0.273) |
| Frequency of physical activity (1=high, 2=low) | 0.064 (0.002, 0.127) |
| Current cigarette smoking (1=yes, 2=no) | -0.086 (-0.149, -0.025) |
| Somatic (R^2^ = 0.171, p<0.001) | |
| Self-rated health (1=poor, 2=good) | -0.320 (-0.386, -0.254) |
| Sex (1=woman, 2=man) | -0.150 (-0.212, -0.087) |
| Anxiety (R^2^ = 0.128, p<0.001) | |
| Sex (1=woman, 2=man) | -0.206 (-0.271, -0.143) |
| Self-rated health (1=poor, 2=good) | -0.228 (-0.295, -0.160) |
| Current cigarette smoking (1=yes, 2=no) | -0.083 (-0.146, -0.019) |
| Social (R^2^ = 0.118, p<0.001 ) | |
| Sex (1=woman, 2=man) | -0.118 (-0.183, -0.054) |
| Frequency of physical activity (1=high, 2=low) | 0.084 (0.019, 0.149) |
| Self-rated health (1=poor, 2=good) | -0.257 (-0.325, -0.189) |
| Depression (R^2^= 0.127, p<0.001) | |
| Sex (1=woman, 2=man) | -0.107 (-0.171, -0.042) |
| Current cigarette smoking (1=yes, 2=no) | -0.100 (-0.165, -0.036) |
| Self-rated health (1=poor, 2=good) | -0.272 (-0.340, -0.203) |
| The second year of observation T2(2022/2023) | |
| Overall GHQ (R^2^ =0.204, p<0.001) | |
| Sex (1=woman, 2=man) | -0.203 (-0271, -0.134) |
| Current financial situation (1=poor, 2=good) | -0.116 (-0.186, -0.047) |
| Self-rated health (1=poor, 2=good) | -0.330 (-0.401, -0.260) |
| Frequency of physical activity (1=high, 2=low) | 0.078 (0.009, 0.147) |
| Somatic (R^2^ = 0.173, p<0.001) | |
| Sex (1=woman, 2=man) | -0.180 (-0.249, -0.112) |
| Self-rated health (1=poor, 2=good) | -0.322 (-0.392, -0.252) |
| Declared chronic disease (1=yes, 2=no) | -0.071 (-0.140, -0.002) |
| Anxiety (R^2^ = 0.159, p<0.001) | |
| Sex (1=woman, 2=man) | -0.230 (-0.299, -0.161) |
| Current financial situation (1=poor, 2=good) | -0.118 (-0.188, -0.049) |
| Self-rated health (1=poor, 2=good) | -0.250 (-0.321, -0.179) |
| Social (R^2^ = 0.116, p<0.001 ) | |
| Sex (1=woman, 2=man) | -0.108 (-0.178, -0.037) |
| Current financial situation (1=poor, 2=good) | -0.086 (-0.157, -0.015) |
| Self-rated health (1=poor, 2=good) | -0.248 (-0.320, -0.175) |
| Frequency of physical activity (1=high, 2=low) | 0.119 (0.048, 0.190) |
| Depression (R^2^= 0.115, p<0.001) | |
| Sex (1=woman, 2=man) | -0.110 (-0.182, -0.038) |
| Current financial situation (1=poor, 2=good) | -0.133 (-0.205, -0.060) |
| Self-rated health (1=poor, 2=good) | -0.263 (-0.335, -0.190) |

Legend: CI, Confidence Interval. R^2^, determination of the model. p, the significance of the multivariable regression model.
