# Supplementary material for: Association between chronic health problems and quality of life in medical students. Results of the POLLEK cohort study
Source: Front Public Health. 2026 May 29;14:1824586. doi: 10.3389/fpubh.2026.1824586 (PMC13259856; doi:10.3389/fpubh.2026.1824586)
Supplement: Supplementary file 3 [file Data_Sheet_1.pdf]

Association Between Chronic Health Problems and Quality of Life in Medical Students. Results of the POLLEK Cohort Study

Szemik S., Pilarska-Kaleta A., Kowalska J., Kowalska M.

Medical students with chronic diseases constitute a particularly vulnerable subgroup of young adults, exposed to various health risks.

Previous findings suggest that medical students with chronic illnesses experience greater deterioration in quality of life (QoL) compared with their healthy peers.

Aim: To validate earlier observations from the POLLEK study regarding the relationship between quality of life, health status, and the prevalence of chronic diseases among medical students during a two-year follow-up.

Observation period: 2020/2021–2022/2023

T1		T2
First year	Longitudinal cohort study (POLLEK) Medical University of Silesia in Katowice	Second year
N = 887		N = 705

Questionnaire included:

- Quality of life (WHOQOL-BREF)
- Hazardous alcohol use (AUDIT)
- General health status (GHQ-28)
- Lifestyle indicators
- Sociodemographic characteristics

Students were divided into two groups:

- **DCD** – Diagnosed chronic diseases
- **NDCD** – No declared chronic diseases

Prevalence of diagnosed chronic diseases

T1 First year	T2 Second year
24.7% (N = 219)	28.9% (N = 204)

Higher overall QoL scores in students without chronic diseases

T1 and T2: $p < 0.001$

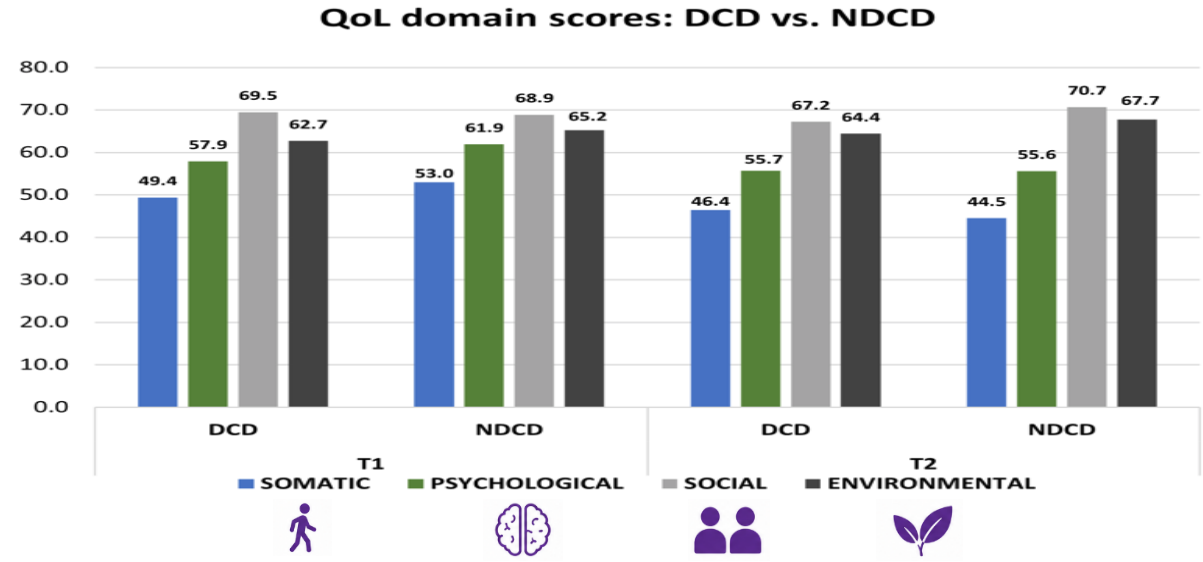

Chronic diseases are relatively common among medical students.

They are associated with poorer quality of life, worse self-rated health, and lower psychological well-being.

Students with chronic diseases report consistently lower quality of life, more somatic complaints, and higher levels of anxiety and depressive symptoms.

These differences persist over time during the early years of medical education.

- Systematic monitoring of students' well-being
- Early, tailored support
- Health-focused interventions and coping support
- Appropriate academic accommodations

POLLEK – Polish medical students' longitudinal study; QoL – quality of life; WHOQOL-BREF – World Health Organization Quality of Life – BREF; AUDIT – Alcohol Use Disorders Identification Test; GHQ-28 – General Health Questionnaire – 28; DCD – diagnosed chronic diseases; NDCD – no declared chronic diseases
